# Novel theory of mind task demonstrates representation of minds in mental state inference

**DOI:** 10.1038/s41598-022-25490-x

**Published:** 2022-12-07

**Authors:** Emily L. Long, Hélio Clemente Cuve, Jane Rebecca Conway, Caroline Catmur, Geoffrey Bird

**Affiliations:** 1 Department of Experimental Psychology, University of Oxford, Anna Watts Building, Woodstock Rd, Oxford, OX2 6GG UK; 2 Institute for Advanced Study in Toulouse, Université Toulouse 1 Capitole, 31080 Toulouse Cedex 06, France; 3 School of Psychology, University of Galway, Galway, H91 TK33 Ireland; 4 Department of Psychology, Institute of Psychiatry, Psychology & Neuroscience, King’s College London, De Crespigny Park, Denmark Hill, London, SE5 8AF UK

**Keywords:** Psychology, Human behaviour

## Abstract

Theory of mind (ToM), the ability to represent the mental states of oneself and others, is argued to be central to human social experience, and impairments in this ability are thought to underlie several psychiatric and developmental conditions. To examine the accuracy of mental state inferences, a novel ToM task was developed, requiring inferences to be made about the mental states of ‘Targets’, prior participants who took part in a videoed mock interview. Participants also made estimates of the Targets’ personality traits. These inferences were compared to ground-truth data, provided by the Targets, of their true traits and mental states. Results from 55 adult participants demonstrated that trait inferences were used to derive mental state inferences, and that the accuracy of trait estimates predicted the accuracy of mental state inferences. Moreover, the size and direction of the association between trait accuracy and mental state accuracy varied according to the trait—mental state combination. The accuracy of trait inferences was predicted by the accuracy of participants’ understanding of trait covariation at the population level. Findings are in accordance with the Mind-space theory, that representation of the Target mind is used in the inference of their mental states.

## Introduction

Theory of mind (ToM) is typically defined as the ability to represent one’s own mental state, as well as the mental states of others^1^. Mental states themselves can be defined as propositional attitudes^2^, such as “Sally believes that the marble is in the basket”—in which Sally (the Target of the inference) is thought to hold an attitude (in this case, a belief in the truth of) a proposition (the marble is in the basket). ToM, then, may be considered the ability to infer and represent one’s own and others’ propositional attitudes. Individual differences in ToM can be considered along two dimensions: the propensity to represent mental states, and the accuracy of those mental state representations^3^.

Questions as to the propensity to engage in mental state representation are highly relevant to the development of social cognition in infancy and childhood^4–7^, or when explaining the features associated with certain clinical conditions^8,9^. Propensity may also explain some individual differences in ToM seen in neurotypical adults, who are thought to be prolific in their ToM use^10,11^. However, variance in the accuracy of mental state inferences is typically considered to be the defining feature of individual differences in theory of mind in neurotypical adults^12^. It is problematic therefore, that tests of mental state inference accuracy have been criticised on a number of levels. Some of these criticisms refer to disagreement as to what is considered a mental state, for example some tests have been criticised on the basis that they measure recognition of facial emotion rather than mental states^13^. Other tests have been criticised on the basis that performance may not reflect mental state inference^5,14^, that they are insensitive when used with neurotypical adults or older neurotypical children due to ceiling effects^15,16^, or that inappropriate control conditions are used^17,18^. Surprisingly, given that tests are measuring the accuracy of mental state inferences, existing tests are rarely criticised on the basis that they lack an objective ground-truth from which to assess the accuracy of mental state inferences.

Ground-truth is a term used to refer to true, objective data that is used as a baseline to assess the performance of an algorithmic or psychological process^19–21^. Crucially, such data is obtained directly, rather than through another inferential process. In the case of measuring ToM accuracy, it is seemingly a basic requirement to know the correct answer when assessing accuracy. Despite this, the vast majority of tests do not offer ground-truth, instead they adopt one of two or three common strategies. First, participants may be exposed to a story, cartoon, or movie designed to invoke attribution of a specific mental state to a character. In these tests it is hard to know what the ground-truth is: for example, if watching a movie, am I accurate if I infer what the script says the character’s mental state should be, or if I infer the mental state of the actor playing the character? If I am instructed to infer the character’s mental state then whether I make the ‘correct’ inference depends on whether the actor depicts the mental state in a way that leads me to infer the author’s intended mental state, or whether the situation prompts the same inference in me as the person who wrote the script. Second, stimuli may be used that describe a situation with one or more protagonists, and participants must make an inference about the character’s mental state. Accuracy in these tests is defined relative to the norm—if you make the same inference as a group of neurotypical adults you are deemed to be correct, if not, you are deemed to be incorrect^12^. A third group of tests is very similar, but these use logic to derive the ‘correct answer’^22^. Tests falling into the second and third categories also do not have an objectively correct ground-truth. The characters, by virtue of being fictional, do not actually have mental states, and therefore ‘accurate’ really means typical (you make the same mental state inference as others), or logical (as defined by the test authors). Since the characters do not actually have mental states, judging whether their mental states are accurately inferred is impossible.

As far as we are aware, there is only one group of tests that can be described as having an objectively correct answer (i.e., tests for which the ground-truth is known), and these are tests of visual perspective taking. In such tests, participants are asked to judge whether another character, occupying a different viewpoint from the participant, can see certain objects, or to judge how objects appear from the character’s viewpoint. Although these tests have an objectively correct answer, they have been criticised on the basis that representing a character’s viewpoint does not qualify as representing a mental state^23^, and, perhaps more importantly, that representing what the character sees is not necessary in these tasks^24,25^.

Other fields within cognitive science have successfully overcome this ground-truth problem. For example, tests of facial emotion recognition used to utilise artificial stimuli in which actors adopted facial expressions of emotion with very low ecological validity and no ground-truth beyond the expression the actors were asked to convey. In contrast, current tests typically use stimuli derived from a process whereby emotions are induced in participants and their facial expressions recorded while they report how they feel. These ratings are used as the ground-truth when the recorded facial expressions are subsequently used as experimental stimuli^26^. We decided to adopt a similar approach in developing a new test of the accuracy of mental state inference, the Interview Task.

Briefly, the Interview Task involves participants watching recordings of real-life, mock job interviews involving an Interviewer and Candidate. At various points, the recordings are paused and participants asked to infer the interview participants’ (‘Targets’) mental states (e.g., How would the Candidate rate their performance in the interview? To what extent does the Interviewer think that they put the Candidate at ease?). Crucially, when the interviews were being recorded, the Candidate and Interviewer were asked these questions and reported their mental states, providing a ground-truth against which the accuracy of participant mental state inferences can be judged.

Measurement issues aside, a psychological framework within which individual differences in adult ToM (specifically mental state inference accuracy) can be interpreted is also necessary. Traditionally this has not been available, as ToM was thought to be subserved by a dedicated psychological module, instantiated in a particular neural circuit including the medial prefrontal cortex and temporoparietal junction^27,28^. Under this modular view, individuals either can, or cannot, represent mental states—it does not address how (or the ways in which) some individuals may be better than others at ToM^29^. Of course, non-specific cognitive abilities, such as language^30^ or executive function^31^, can affect performance on any cognitive test that may utilise these abilities, including tests of ToM ability. Newer models detail the psychological, neurological, and/or computational processes involved in mental state inference^32–34^ and so provide for understanding of individual differences. One such framework, Mind-space, aims to outline the sources of individual differences in the accuracy of mental state inferences. Under this framework individuals use (at least) two pieces of information when making mental state inferences: the situation a Target is in, and the type of mind they have^35^. It is suggested that individuals construct a multi-dimensional ‘Mind-space’ in which each dimension represents a way in which minds can be individuated (memory capacity, IQ, personality dimensions, etc.), and the space represents the covariance between dimensions (e.g., the positive correlation between verbal fluency and working memory). As such, dimensions may be non-orthogonal, such that if there is a positive correlation between conscientiousness and extraversion in the general population, for example, then the space would be constructed such that those who are more conscientious will be higher on extraversion. A ‘Target’ mind (i.e., the mind of the individual whose mental states I am trying to infer) is represented as a location within Mind-space, and the likelihood of a particular mental state is inferred on the basis of the situation the Target is in and the location of the Target’s mind within Mind-space. For example, if the Target is lying in bed in the dark and the room suddenly gets cold and you are asked to predict the Target’s mental state, if you consider them to be superstitious then you might predict that they will believe that a ghost is in the room, whereas if you consider them to be a sceptic you might predict that they will believe that a window has blown open. Under the Mind-space framework, therefore, the following are all sources of individual differences in the accuracy of mental state inferences: (1) The accuracy of Mind-space (particularly of any covariance between dimensions); (2) The ability to locate accurately a Target mind within Mind-space; (3) The ability to combine diagnostic information about the situation and the location of the Target in Mind-space to infer accurately their mental state; (4) The propensity to consider position in Mind-space before making a mental state inference.

There is currently little empirical work testing the predictions of the Mind-space framework, but Conway and colleagues^36^ showed that individuals consider the mind of the Target individual, and of other social agents who have influence over the Target’s mental states, when attempting to infer Targets’ mental states. They also found that those with better models of how minds vary (i.e., those with a more accurate Mind-space) were better able to locate Target minds within Mind-space and were more likely to make typical mental state inferences. In Conway and colleagues’ study, mental state inference ability was measured using the Movie for the Assessment of Social Cognition (MASC^12^). The MASC has the twin advantages of using more ecologically valid stimuli (participants watch a movie of a social interaction) and being sensitive to variance within the adult neurotypical population. It does lack ground-truth, however, as correct answers are determined by the movie script and consensus scoring—with accuracy determined by what a sample of neurotypical adults believe to be the correct answer. Here, therefore, instead of the MASC, we use the Interview Task to assess some central claims of the Mind-space theory, particularly in relation to the accuracy of mental state inferences.

Specifically, participants completed the Interview Task and the Personality Pairs Task (PPT). The Interview Task allows two abilities relevant to Mind-space to be tested. The first, as previously described, is the accuracy of mental state inferences—these can be assessed against ground-truth data provided by Candidate and Interviewer reports of their mental states. The second is the ability of participants to locate Target minds in Mind-space. Each Candidate and Interviewer completed measures of personality and IQ. Participants completing the Interview Task are asked to estimate the traits of Candidates and Interviewers, and thus their ability to estimate a Target’s traits (i.e., the ability to locate a Target mind within Mind-space) can be assessed. The other task, the PPT, is designed to test the accuracy of Mind-space, specifically a participant’s understanding of how personality traits co-vary. In the PPT, participants are asked to estimate the correlation between traits on six personality dimensions in the general population. These estimated correlations can then be compared to inter-trait correlation values obtained from large independent samples to determine the accuracy of an individual’s Mind-space.

Using the Interview Task and the PPT, three hypotheses from the Mind-space framework can be tested. First, that the accuracy with which participants can place Targets in Mind-space will predict the accuracy of their mental state inference (both measured using the Interview Task). Under the Mind-space framework participants combine information about the situation the Target is in and the type of mind they have in order to calculate the probability they will have specific mental states. The better able they are to locate Targets within Mind-space, the better able they should be to infer mental states accurately (given that they correctly understand the relationship between location in Mind-space and the probability of specific mental states). We would expect, based on findings from Conway and colleagues^36^, that the accuracy of mental state inference will be predicted not only by the accuracy with which participants locate the Target of inference, but also the accuracy with which they locate the Target’s counterpart (i.e., the Interviewer when considering the Candidate’s mental state, or vice versa), because the counterpart will likely impact the Target’s mental states in a trait-dependent fashion. Second, that participants will update their estimate of the likelihood of specific mental states as their estimate of the Target’s location within Mind-space changes (and that the size of this effect will vary across mental state inferences due to varying strengths of relationships between specific Mind-space dimensions and specific mental state inferences). If participants incorporate the location of a Target mind in Mind-space when assessing the probability the Target will have specific mental states, then it follows that if the location of the Target moves in the participant’s Mind-space, then the probability of specific mental states will change. The size of the effect on mental states is likely to vary according to the participant’s model of how the dimension(s) along which the Target has moved in their Mind-space relates to particular mental states. For example, if I realise that a Target is much more extrovert than I previously thought they were, then I might judge the likelihood that they will believe that they will be bored if they are on their own much higher than I did previously. However, my estimate of the likelihood that they believe that they will be hungry is likely to be unaffected. Again, this hypothesis can be tested using the Interview Task. Third, that the accuracy of Mind-space (measured using the PPT) will be associated with the accuracy with which Targets are located within Mind-space (measured using the Interview Task). This relationship, first reported by Conway and colleagues^36^, is consistent with the idea that in order to build an accurate model of how traits co-vary in the population, you must first be good at reading those traits in others. Similarly, it would be expected that an accurate model of population-level co-variation would improve the ability to infer a Target’s traits (e.g., when direct information is present about one trait but not about another, related, trait). Finding the predicted association would thus further support the notion that at least one of these mechanisms occurs in mental state inference.

## Methods

### Participants

61 participants volunteered to participate. As no equivalent data or analyses on which to base power calculations exist, we recruited as many participants as possible with available funds to maximise the power of the study. However, a simulation-based power calculation conducted on the basis of effects observed in an independent dataset (N = 25) indicates that the present study has a power of > 99% (for full details, see Supplementary Information, section S2). Participants were recruited through advertisements placed across University of Oxford locations and online, and through a departmental online research participation platform. All participants were compensated with either money or course credits. One participant was excluded for failing two attention checks, one was excluded due to technical difficulties and four were excluded for not completing the Personality Pairs Task, leaving 55 participants (38 female, M_AGE_ = 19.91, SD_AGE_ = 1.45, age range = 17–25) for analysis. All participants provided informed consent and the study was approved by the University of Oxford Central University Research Ethics Committee and followed the principles of the Declaration of Helsinki.

Eight participants (7 female, M_AGE_ = 37.82, SD_AGE_ = 18.11, age range = 21–65) were recruited to be videorecorded for the stimuli. Participants in stimulus production were recruited through advertisements across University of Oxford locations and online, as well as through a community centre located in Tewkesbury, UK. All participants provided informed consent and were given monetary compensation.

### The Interview Task

#### Stimulus development

Each participant who was recruited for videorecording completed the HEXACO-60^37^ personality inventory, which provides measurements of the following traits: honesty-humility, emotionality, extraversion, agreeableness, conscientiousness, and openness-to-experience^38^. Descriptions of these domain-level scales can be found at www.hexaco.org. Participants also completed the vocabulary and matrix reasoning portions of the WASI-II^39^ as an estimate of their full-scale IQ. These participants then took part in mock job interviews, with each participant taking part in one mock interview in the role of the Interviewer and in another as the Candidate, remaining with the same partner for both interviews. Interviewers were given a list of questions to be asked but were free to ask any follow-up questions they wished. Halfway through each interview and at the end, Interviewers and Candidates were asked to report, in private, their mental states (see Supplementary Information, section S1.1., for the mental state questions answered) along a given set of dimensions; these asked about their perception of their and their counterpart’s performance, their perception of what their counterpart thought of them, and their perception of their counterpart’s traits and emotions. Participants were made aware that, unlike their written profiles and video recordings, only the researchers would have access to their reported mental states and were asked to acknowledge their understanding of this prior to participation. Responses were given along continuous sliders, with the extremes labelled appropriately for the question. For example, the Candidate was asked “How would you rate your performance in the interview?” with the slider ends labelled as “Very poor” to “Excellent.” These reports thus constitute ground-truth data about the actual mental states of the Targets to be compared to inferences made by participants while watching these videos. Each slider response was converted to a numerical value on a 0–100 scale, allowing them to later be subtracted from participant responses on the same scale.

Interviewers and Candidates were also asked to complete a written profile about themselves in which they provided information about how they perceive their communication and leadership skills, their thinking style, and their interpersonal and technical skills.

#### Procedure

Before watching each of the four mock interviews, participants read the written profiles of the Interviewer and Candidate. In an effort to ensure that participants updated the position of the Interviewer and Candidate in their Mind-space, half of the profiles were switched, so that the written profile was not of the person the participant was about to see in the videorecording. This resulted in three types of trial—‘matched’ trials, where true profiles were presented for both Targets; ‘half’ trials, in which one Target had a mismatched profile; and ‘mismatched’ trials, where both Targets were associated with incorrect profiles. Each participant viewed one pair of matched Targets, two pairs of half matched Targets, and one pair of mismatched Targets.

Participants were asked to judge the personalities (along the six HEXACO dimensions: honesty-humility, emotionality, extraversion, agreeableness, conscientiousness, openness-to-experience^38^) and IQs (relative to the population average) of the Interviewers and Candidates, and then to infer their mental states—where the mental states they were asked to infer matched the mental state questions answered by Interviewers and Candidates (see Supplementary Information, sections S1.1. and S1.2., for the mental state questions answered by Targets and participants respectively). Again, responses were given along continuous sliders, with the extremes labelled appropriately for the question. For example, participants were asked “How would the candidate rate their performance in the interview?” with the slider ends labelled as “Very poor” to “Excellent.” For mental state questions, each slider response was converted to a numerical value on a 0–100 scale, thus making them comparable to the responses of the Targets. For personality trait judgements, each slider response was converted to a numerical value on a 0–100 scale, and responses were then transformed to a 1–5 scale, so that these responses were comparable to the HEXACO-60 trait scores of the Targets. Similarly, IQ judgements were converted to a 70–130 scale to allow comparison to IQ scores obtained on the WASI-II. Participants were asked to do this before watching the interview (on the basis of the written profiles), half-way through watching the interview, and after watching the whole of the interview.

### The Personality Pairs Task (PPT)

To test the accuracy of participants’ Mind-space, participants were given the Personality Pairs Task (PPT^36^). This task involves answering questions of the form “On average, how likely is it that someone who, *when s/he is in a group of people, is often the one who speaks on behalf of the group,* would also *want people to know that s/he is an important person of high status*?” The items were taken from the HEXACO-60 personality questionnaire^37^. Observers had to indicate the likelihood of the two traits co-occurring on a scale of − 100 to 100, with − 100 labelled as “Very unlikely” and 100 labelled as “Very likely”. There were two pairs presented for every combination of the six HEXACO personality dimensions^38^.

### Testing procedure

Gorilla Experiment Builder (www.gorilla.sc) was used to create and host the experiment^40^. After viewing the information sheet and providing consent, participants completed the PPT. They then completed the Interview Task in one of four streams. Each stream had a different order of presentation for the videos, and it was randomly determined which videos and profiles would be mismatched for each stream, and thus which videos would appear as which trial type. Therefore, all participants saw all videos (in one of four orders), but the profiles matched to the videos followed four possible combinations. Participants were pseudo-randomly allocated to one of the four streams, such that the number of participants who completed each stream was balanced. The experiment took approximately one and a half hours to complete.

### Analysis strategy

#### PPT

The PPT was scored in the same manner as Conway et al.^36^: namely, estimates were divided by 100 to make them comparable to a correlation coefficient, then compared to actual inter-trait correlation values from a sample of 2868 taken by Lee and Ashton^41^. Following Conway and colleagues, the absolute difference between estimates and actual values was taken for each question and the mean of these absolute differences was used as the PPT error score, such that a higher value indicates greater error and thus a less accurate Mind-space.

#### Interview Task

An update was calculated as the difference in an estimate between two consecutive judgements (i.e., from before the video to the mid-point, or from the mid-point to the end) by subtracting the earlier judgement from the later judgement. An update of zero indicates no change, whilst non-zero datapoints indicate an update to the given rating in the given direction (i.e., positive values indicate an increase, whilst negative values indicate a decrease). Error was calculated as the participant’s estimate minus the Target’s ground-truth report (see Supplementary Information, sections S1.1. and S1.2., for the mental state questions answered by Targets and participants respectively). Errors are taken from reports half-way through the mock interview and at the end, as accuracy is not meaningful for trials in which participants assess Targets on the basis of a mismatched profile, prior to observing the true Target. An error of zero indicates a perfect estimate, whilst non-zero datapoints indicate an error in the given rating in the given direction (i.e., positive values indicate an overestimate, whilst negative values indicate an underestimate).

Outlier datapoints were defined as those where trait or mental state judgement updates or errors were more than 1.5 times the interquartile range away from the upper or lower quartile for the specific measure, and such datapoints were removed prior to analysis. Because the IQ estimates were on a markedly different scale to HEXACO trait estimates, and because scaling these estimates would distort the variance structure and affect interpretability, IQ estimates were omitted from primary analysis. Analyses of IQ data are available in the Supplementary Information (section S5) and show the same pattern of results as the personality trait analyses. Linear mixed-effects models were used to analyse the Interview Task data. An explanation of these models and the use of the lme4 package for R^42^ is given by Bates and Mächler^43^.

Prior to testing our hypotheses, we investigated the effect of our trial types (‘matched’, ‘half’, and ‘mismatched’ trials) on trait updates by fitting and comparing models which used trial type and time to predict the absolute magnitude of trait updates from prior to the interview to half-way through the interview, and from half-way through the interview to the end of the interview. Note that the mismatching was performed in order to ensure that participants updated their estimates of Target personality but updating was also expected in the other trial types.

Then, model selection was performed by comparing statistical models for each of our three hypotheses. First, we assessed whether the accuracy of participant estimates of Targets’ HEXACO traits predicted the accuracy of mental state inference and examined the variance in this effect across combinations of traits and mental state inferences. As we hypothesised that the effect of the accuracy of each trait estimate may differ according to participant beliefs about its relationship with specific mental states, the variance in the random effects is an important model output. Second, we examined updates in trait estimates and specific mental state inferences, again inspecting the variance in any relationship according to the trait in question and the specific mental state inference being made. Third, we investigated the extent to which the accuracy of Mind-space (measured by the PPT) predicted the accuracy with which Targets were located, i.e., the accuracy of trait estimates. Full details of the models considered in the selection process can be found in the Supplementary Information (section S3).

Model comparison was conducted using the anova function from the lmerTest package^44^ which applies a log likelihood ratio test and provides AIC and BIC values, which are model fit criteria that allow assessment of explanatory power relative to model complexity and generally prefer the most parsimonious model^45^. In the case of AIC and BIC values, a reduction of six or more points was deemed to indicate a better model. An alpha of 0.05 was used to determine whether a more complex model significantly outperformed a less complex model according to the log likelihood ratio test. Models were nested, such that more complex models always contained all terms from the less complex models. All three criteria agreed on the best model for all of our primary hypotheses. For the one case in which they did not agree, when testing the effect of trial type, the AIC was used. This is due to the fact that, given the categorical nature of the variable, several parameters were estimated for the trial type variable, and the BIC may therefore have been too conservative^46^. Inferences drawn from the models make use of the summary function in lmerTest, which provides estimates for the variance in the random effects and conducts significance tests of fixed effects using Satterthwaite’s method for estimating degrees of freedom.

Testing the hypothesis that the relationship between trait updates or accuracy, and mental state updates or accuracy, is dependent upon the trait-mental state combination in question relies on examination of the variance of random slopes, specifically the variance of the slope of trait update/accuracy based on trial (i.e., trait-target-mental state combination). Given the lack of an established way to test the significance of random slope variance, parameter distributions were constructed to explore the likelihood of finding the observed level of variance from the best-fitting model were there no systematic relationship between specific traits and mental states across participants and stimuli. Specifically, data were grouped by participant, stimulus and mental state and the trait update/accuracy values were shuffled (i.e., sampled without replacement) within each group, effectively dissociating the trait estimate from its trait label. An equivalent model to our final model was then fitted to the shuffled dataset. This process was repeated 1000 times for each analysis, allowing parameter distributions to be constructed. If, contrary to our hypothesis, there were no systematic relationship between specific trait estimates and specific mental state inferences across participants and stimuli, then comparison of the values obtained from our models with the correct trait labels to the derived parameter distributions would reveal comparable parameter values.

The analyses described above seek to determine whether the accuracy of mental state inference is related to the accuracy of trait estimates and, similarly, whether updates in mental state inference are related to updates in trait estimates. As established in the Introduction, however, one would expect different trait estimates to be differentially related to specific mental state inferences. For example, it might be that a Candidate who is high in trait emotionality is thought, by participants, to be likely to give a high rating endorsing the statement that “the interviewer thinks they are nervous” and a low rating in response to the question “How confident are you that you would get the job?”. In this case, then, Candidate emotionality would have a positive relationship with the former mental state, and a negative relationship with the latter. However, these specific relationships should be systematic across participants and across Targets. In general, participants will have learned the relationships between specific traits and mental states in broadly similar social environments and these learned relationships should be assumed to hold for all observed Targets.

The statistical methods described above therefore examine whether the relationships between specific traits and mental states, in both accuracy and updates, vary systematically. Specifically, the models used allow the fitted slopes to vary both by the trait-target-mental state combination (i.e., trial) and by participant. If the above description of systematic variation holds, it would be expected that the variance accounted for by the varying trial slopes would be greater, and the model residual lower, in our observed data than in simulated data in which trait labels have been dissociated from their accompanying inferred values through randomising trait labels. The simulation described above provides a direct means of exploring this. Having constructed distributions of parameter values by simulation, we were able to compare the observed model statistics to what would be expected if mental state inferences did not vary systematically with specific trait estimates. As the only aspect of the underlying data which changes in these simulations is the trait labelling, differences in model parameters between the observed and simulated models would indicate the systematic relationships we predict. We would expect the greatest parameter differences to emerge in the variance of the random slope on the trial grouping term and the residual, as shuffling trait labels should decrease the amount of variance explained by allowing the relationships between trait update/error and mental state update/error to vary by trial. To examine this, we computed z-scores for each of our observed parameter values relative to the distribution of the same parameter across the simulated datasets and descriptively compared the resulting z-scores for different parameters.

## Results

**Figure 1:**
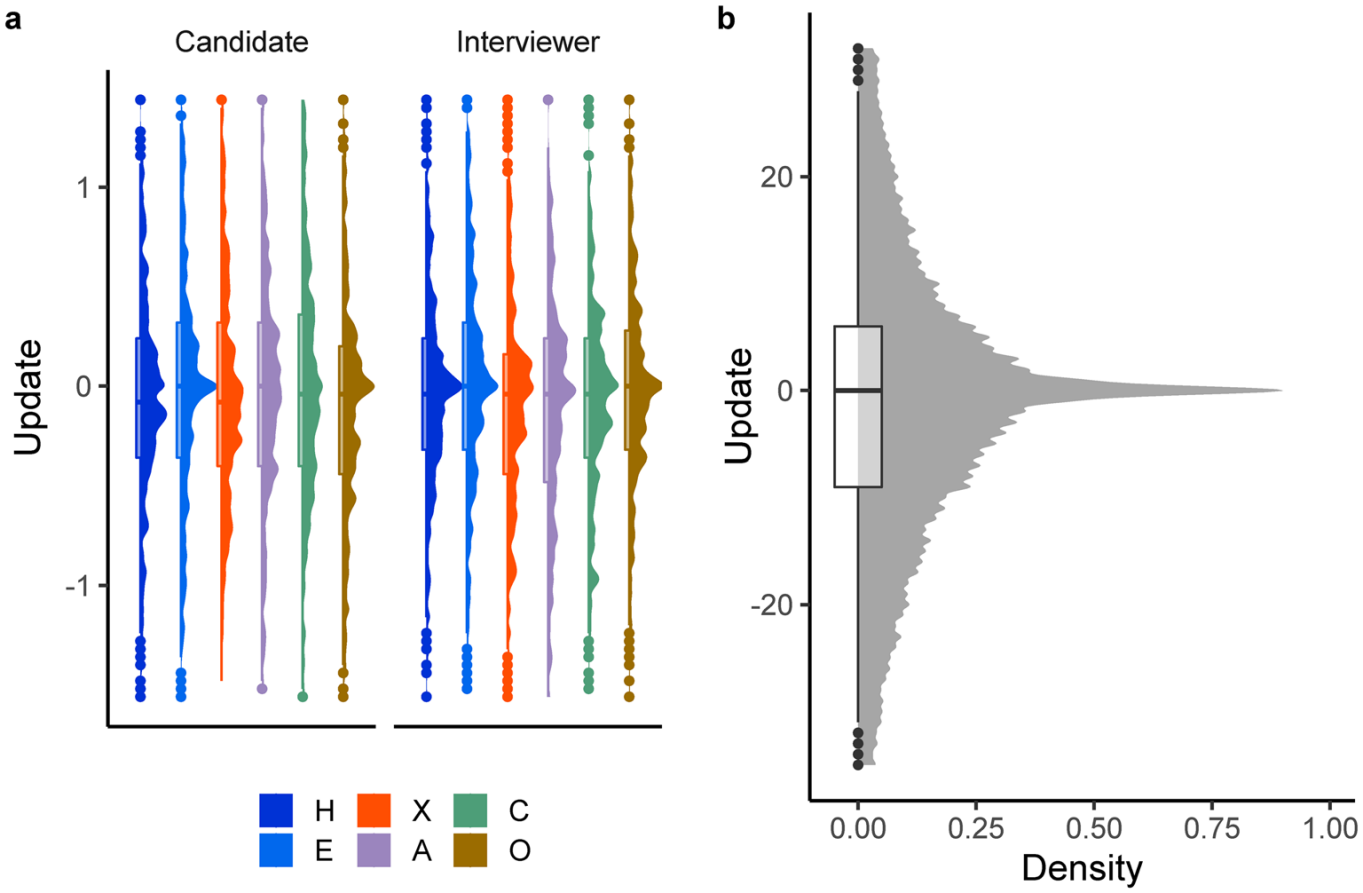
Density plots and boxplots of the distributions of updates in trait estimates and mental state inferences for the Interview Task. An update was calculated as the difference in an estimate between two consecutive judgements (i.e., from before the video to the mid-point, and from the mid-point to the end). An update of zero indicates no change, whilst non-zero datapoints indicate an update to the given rating in the given direction (i.e., positive = increase, negative = decrease). (Panel **a**) The distribution of updates for each trait for each Target ‘type’ (i.e., Interviewer or Candidate). H = honesty-humility, E = emotionality, X = extraversion, A = agreeableness, C = conscientiousness, O = openness to experience. (Panel **b**) The distribution of mental state updates.

**Figure 2:**
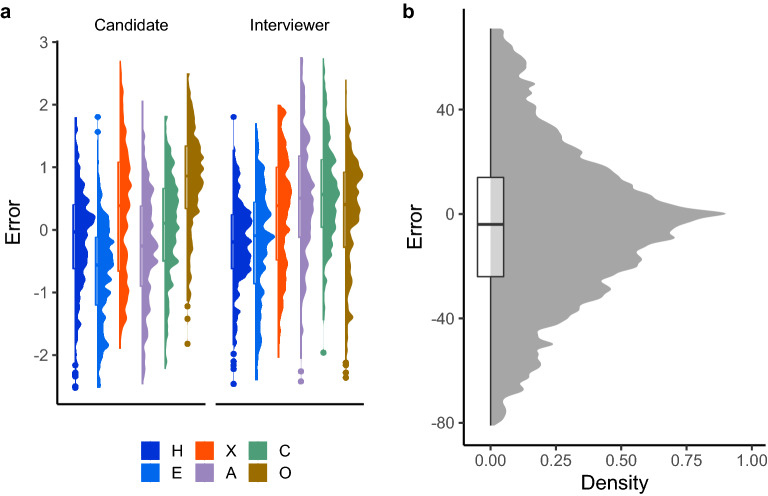
Density plots and boxplots of the distributions of errors in trait estimates and mental state inferences for the Interview Task. Error is calculated as the participant’s estimate minus the Target’s ground-truth report. Errors are taken from reports half-way through the interview and at the end. An error of zero indicates a perfect estimate, whilst non-zero datapoints indicate an error in the given rating in the given direction (i.e., positive = overestimate, negative = underestimate). (Panel **a**) The distribution of errors for each trait for each Target ‘type’ (i.e., Interviewer or Candidate). H = honesty-humility, E = emotionality, X = extraversion, A = agreeableness, C = conscientiousness, O = openness to experience. (Panel **b**) The distribution of mental state errors.

Descriptive statistics for the Interview Task and illustrations of the distributions of updates to trait estimates and mental state inferences are presented in Fig. 1, and the same information is provided for the accuracy of trait and mental state estimates in Fig. 2. Note that all Interview Task estimates were reported by participants on a scale of 0–100, but that trait estimates were transformed to a 1–5 scale, to match the scale of the HEXACO-60 results of the Targets. For the PPT, error scores had a mean of 0.32 and a standard deviation of 0.09.

### Mismatching intervention

The mismatching of some profiles and videos was intended to ensure updates to trait estimates, to enable analysis of how these updates influenced mental state inferences. It was expected that trait estimate updates would be most likely to occur in the first set of judgements made by participants after having seen the Targets (i.e., judgements made halfway through watching the interview). To examine this, we fitted a model using trial type and timepoint to predict the absolute magnitude of the update in trait estimates, with random intercepts for participant and trait and a random slope for timepoint on both intercepts. The final model formula (in lmer syntax) was thus as follows: mental state update ~ trial type * timepoint + (1 + timepoint|participant) + (1 + timepoint|trial). The model of interest significantly outperformed the null model and a model containing only trial type as a fixed effect (see Supplementary Information section S3 for further details).

There were three possible trial types: matched, half, and mismatched and the matched type was used as the reference level. There were two levels of time: pre-mid (i.e., updates from before the interview to mid-way) and mid-end (i.e., updates from half-way through the interview to the end). There was a main effect of time, such that updates were significantly larger for pre-mid (M = 0.50) than for mid-end (M = 0.38; *B* = 0.15, *SE* = 0.03, *t* (105.62) = 5.85, *p* < 0.001). There was no main effect of trial type and no interactions (all *p*s > 0.088, full details are given in the Supplementary Information, section S4). This indicates that participants tended to change their trait estimates after seeing the first portion of the video regardless of whether the written profile was mismatched, and therefore that the mismatching of profiles was unnecessary. It is clear from the descriptive statistics and the effect of time that updates did occur but were independent of our attempts to induce them. Because of this, the effect of trial type will not be examined further.

In the models that follow, data from all three timepoints (i.e., before, during and after the interview) were used to examine updates. In contrast, accuracy models were fitted only to data collected at the mid-point of the interview and at the end, as it is unreasonable to assess the accuracy of trait and mental state inferences against a mismatched Target prior to observing that Target.

### Accuracy of trait estimates and mental state inferences

The best fitting model for predicting errors in mental state inferences was a maximal model which had errors of trait estimates as a predictor and included random intercepts for participant and trial. In the best fitting model, random slopes were fitted for trait estimate error on both random intercepts. The final model formula (in lmer syntax) was thus as follows: mental state error ~ trait error + (1 + trait error|participant) + (1 + trait error|trial). Full details of model comparisons are given in the Supplementary Information (section S3).

**Figure 3:**
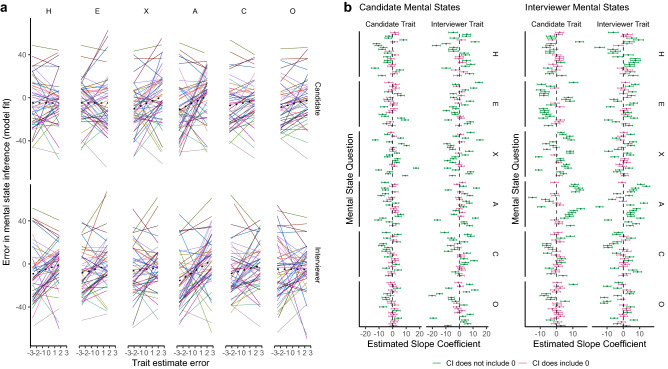
Plots illustrating the model estimates for the relationships between trait estimate errors and mental state inference errors in the Interview Task. (Panel **a**) The specific relationships between errors in estimates of individual traits (given along the top), for specific Targets (given on the right), for different mental states (represented by different colours). The black dotted line represents the overall effect for that trait and Target. The graphs show that the relationships between specific trait errors and specific mental state errors vary such that, for some questions, a trait overestimate might be related to a mental state overestimate, but for others the relationship may be reversed. Similarly, the trait might not be relevant at all to the mental state, resulting in a flat line. (Panel **b**) The estimated slopes for the effect of specific trait estimate errors (given on the right) for specific Targets (given along the top) on specific mental states (represented by separate lines). The estimated slopes for inferences about the Candidate’s mental states are given to the left, those for the Interviewer’s mental states are given to the right. Error bars are 95% confidence intervals and green lines indicate that the confidence interval does not include zero, whilst pink lines indicate that it does**.** This graph therefore further shows that these relationships vary between traits, i.e., each trait has a unique set of mental states which it is related to, each with its own extent and direction, and that both Targets’ minds are considered in mental state inference.

Table 1 gives the full model statistics for this analysis. There was a significant overall effect of trait estimate error on mental state inference error (*B* = 0.89, *SE* = 0.29, *t* (363.32) = 3.09, *p* = 0.002). Importantly, our simulations suggested that the error produced in mental state inference is highly dependent on the mental state being inferred and that the effect of trait estimate error varies systematically between combinations of traits and mental states. These relationships are depicted in Fig. 3. The variance of the trait error random slope obtained from the true, unshuffled data had a z-score of 350.74 relative to the mean and standard deviation of the parameter distribution obtained when the trait error values were shuffled within participant, stimulus and mental state across 1000 iterations. The equivalent z-score for the residual was − 294.88, indicating that the random slope has more predictive power in our observed data than the shuffled data. No other parameter had a z-score larger than 72.10. The full parameter distributions can be seen in the Supplementary Information (section S6). Thus, although relationships between specific traits and mental states vary, relationships are systematic across participants and stimuli.

**Table 1:** Model summary for predicting error of mental state inferences.

Random effects					
Groups	Term	Variance	SD	Correlation	
Trial	Intercept	377.34	19.43		
	Trait error	32.1	5.67	− .04	
Participant	Intercept	8.09	2.85		
	Trait error	1.34	1.16	.26	
Residual		506.01	22.5		

Fixed effects					
Term	Estimate	SE	df	t-value	*p*
Intercept	− 4.76	0.90	556.72	− 5.30	< .001
Trait error	0.89	0.29	363.32	3.09	.002

### Updates of trait estimates and mental state inferences

The best fitting model for predicting updates to mental state inferences was once again a maximal model which had updates to trait estimates as a predictor and included random intercepts for participant and trial. In this model, random slopes were fitted for trait estimate updates on both random intercepts. The final model formula (in lmer syntax) was thus as follows: mental state update ~ trait update + (1 + trait update|participant) + (1 + trait update|trial). Full details of model comparisons are given in the Supplementary Information (section S3).

**Figure 4:**
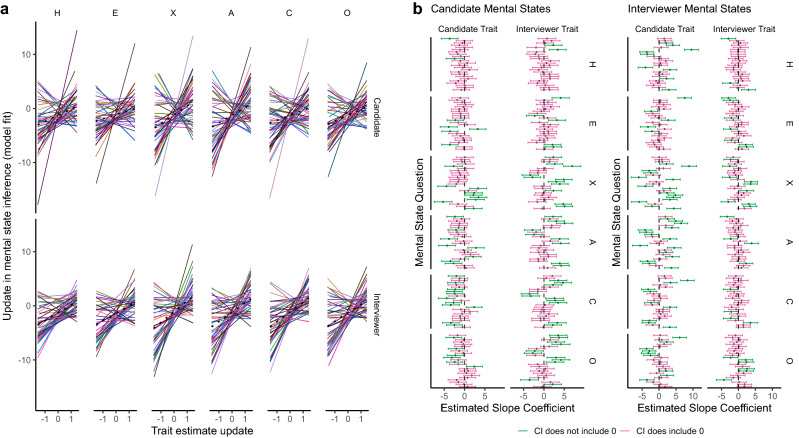
Plots illustrating the model estimates for the relationships between trait estimate updates and mental state inference updates in the Interview Task. (Panel **a**) The specific relationships between updates in estimates of individual traits (given along the top), for specific Targets (given on the right), and different mental states (represented by different colours). The black dotted line represents the overall effect for that trait and Target. The graphs show that the relationships between specific trait updates and specific mental state updates vary such that, for some questions, a trait increase might be related to a mental state inference increase, but for others the relationship may be reversed. Similarly, the trait might not be relevant at all to the mental state, resulting in a flat line. (Panel **b**) The estimated slopes for the effect of specific trait estimate updates (given on the right) for specific Targets (given along the top) on specific mental states (represented by separate lines). The estimated slopes for inferences about the Candidate’s mental states are given to the left, those for the Interviewer’s mental states are given to the right. Error bars are 95% confidence intervals and green lines indicate that the confidence interval does not include zero, whilst pink lines indicate that it does. This graph therefore further shows that these relationships vary between traits, i.e., each trait has a unique set of mental states which it is related to, each with its own extent and direction, and that both Targets’ minds are considered in mental state inference.

Table 2 gives the full model statistics for this analysis. There was a significant overall effect of trait estimate update on mental state inference update (*B* = 1.20, *SE* = 0.21, *t* (89.61) = 5.58, *p* < 0.001). Our simulations suggested that the update observed to mental state inference is highly dependent on the mental state being inferred and that the effect of trait estimate updates varies systematically between combinations of traits and mental states. These relationships are depicted in Fig. 4. The variance of the trait update random slope obtained from the true, unshuffled data had a z-score of 46.10 relative to the mean and standard deviation of the parameter distribution obtained when the trait update values were shuffled within participant, stimulus and mental state across 1000 iterations. The equivalent z-score for the residual was − 45.93, indicating that the random slope has more predictive power in our observed data than the shuffled data. No other parameter had a z-score larger than 10.27. The full parameter distributions can be seen in the Supplementary Information (section S6). Thus, although relationships between specific traits and mental states vary, relationships were again systematic across participants and stimuli.

**Table 2:** Model summary for predicting updates to mental state inferences.

Random effects					
Groups	Term	Variance	SD	Correlation	
Trial	Intercept	2.35	1.53		
	Trait update	5.88	2.43	− .34	
Participant	Intercept	1.71	1.31		
	Trait update	1.82	1.35	− .16	
Residual		163.07	12.77		

Fixed effects					
Term	Estimate	SE	df	t-value	*p*
Intercept	− 1.23	0.19	69.54	− 6.47	< .001
Trait update	1.20	0.21	89.61	5.58	< .001

### Accuracy of Mind-space and trait estimates

To evaluate the effect of the accuracy of Mind-space on the accuracy of trait estimates, we compared models which predicted the absolute error of trait estimates, as there is no reason to believe that PPT score would predict the direction of any error. The best fitting model for predicting absolute error in trait estimate was again a maximal model which used PPT score as a predictor and included random intercepts for participant and the trait being judged. Random slopes were fitted for PPT score on the trait intercept, but not on participant due to the nature of PPT score as a between-subjects variable. The final model formula (in lmer syntax) was thus as follows: absolute trait error ~ PPT error score + (1|participant) + (1 + PPT error score|trial). Full details of model comparisons are given in the Supplementary Information (section S3).

**Figure 5:**
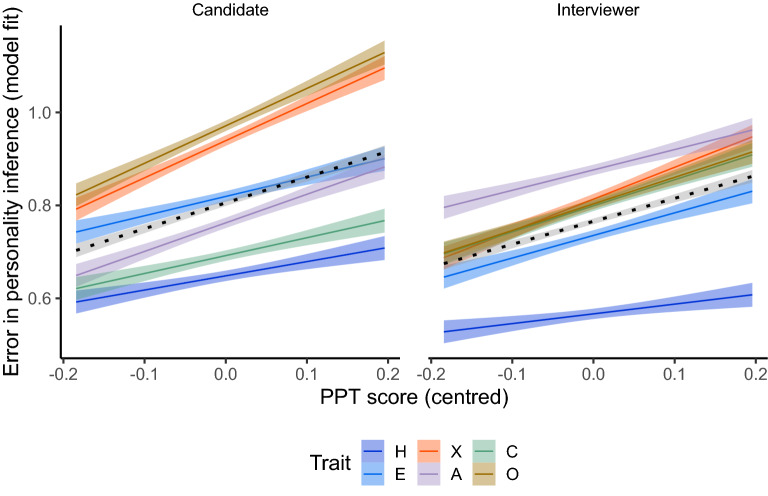
Plot illustrating the model estimates for the relationship between Mind-space accuracy on the PPT and trait inference error on the Interview Task. The graph shows the relationship between centred PPT error score (negative values indicate less error, i.e., better Mind-space accuracy) and trait estimate error for each trait and Target. The black dotted line represents the overall effect for that Target, regardless of trait. Shaded areas show standard errors.

Table 3 gives the full model statistics for this analysis. There was a significant effect of PPT score on absolute trait inference error (*B* = 0.52, *SE* = 0.21, *t* (49.52) = 2.47, *p* = 0.017). This relationship, broken down by the trait being inferred, is depicted in Fig. 5.

**Table 3:** Model summary for predicting error in trait estimates.

Random effects					
Groups	Term	Variance	SD	Correlation	
Trait	Intercept	0.01	0.11		
	PPT error score	0.05	0.24	.64	
Participant	Intercept	0.02	0.13		
Residual		0.29	0.54		

Fixed effects					
Term	Estimate	SE	df	t-value	*p*
Intercept	0.79	0.04	18.56	20.63	< .001
PPT error score	0.52	0.21	49.52	2.47	.017

## Discussion

This study introduced the Interview Task, a novel task designed to assess ToM, specifically the accuracy with which individuals can infer the mental states of others. The task also assesses the ability of individuals to estimate the personality traits of others. Importantly, both mental state inferences and trait estimates could be compared against ground-truth information obtained from the Targets themselves. The Interview Task was used to test three predictions derived from the Mind-space framework. First, that the accuracy with which participants can place Targets in Mind-space will predict the accuracy of their mental state inference. Second, that participants will update their estimate of the likelihood of specific mental states as their estimate of the Target’s location within Mind-space changes (and that the size of this effect will vary across mental state inferences due to varying relationships between specific Mind-space dimensions and specific mental state inferences). Third, that the accuracy of Mind-space will predict the accuracy with which Targets are located within Mind-space.

Results supported all three hypotheses, providing further support for the Mind-space framework and the general principle that, when inferring mental states, individuals consider the mind giving rise to those mental states. Importantly, use of the Interview Task meant that predictions relating to the factors that determine the accuracy of mental state inferences could be tested for the first time, without using the typicality or rationality of mental state inferences as a proxy for accuracy. The Targets of inference, the Candidates and Interviewers who took part in our mock interviews, reported their beliefs about their and their counterpart’s performance, which constitute mental states under the definition of mental states as propositional attitudes^2^. Importantly, whilst tasks which make use of consensus scoring may lead to ‘correct’ answers which reflect the beliefs of the majority, not the beliefs of the Target, accuracy in the present study is assessed relative to the idiosyncratic beliefs of the Target. For example, a Candidate with strong technical skills may not believe themself to have strong technical skills. A consensus-based scoring approach may lead to inferences being judged against the consensus of the Candidate’s true abilities, not against the Candidate’s *belief* about their abilities. As such, accuracy in the present study necessarily reflects the accuracy of mental state inference as accuracy is determined by the Target’s belief, which may not be the case for consensus approaches, especially in cases where the Target themselves has an atypical mind.

Despite the use of a new task, results are in accordance with those of Conway and colleagues^36^ who used the MASC to assess the accuracy (or more properly typicality) of mental state inferences, and showed that the accuracy of Mind-space predicted MASC performance. Furthermore, the present results replicate Conway and colleagues’ finding that mental state inferences are derived from the perceived traits of both the Target of inference and of other social agents with influence over the Target’s mental states. In addition to the use of the Interview Task rather than the MASC, the current study differed from that of Conway et al. in another important way—Mind-space location accuracy was obtained on the basis of much more information about Targets’ behaviour. Conway et al. used ‘thin slices’ of behaviour, where participants watched Targets recite irrelevant sentences for six to nine seconds only. Here, participants were able to observe extended samples of behaviour over two to six minutes. Despite the difference in the amount of information available to participants between studies, in both studies mental state inferences were aided by the ability to locate Targets in Mind-space and predicted by the accuracy of Mind-space.

Furthermore, the current results show that as judgements of the Target’s personality were updated as more information became available, mental state inferences were updated accordingly. This temporal feature of the Interview Task—where participants have the opportunity to update their estimates of Target mental states over time—further differentiates the Interview Task from previous measures of ToM. The mismatching manipulation, which was intended to ensure that participants updated their trait inferences, did not have a significant effect on trait inference updates. Participants’ willingness to update their trait estimates on the basis of the first half of the mock interview, regardless of the validity of pre-existing information, may have been because the videos provided more direct evidence of Target traits, or because participants did not trust information written in an application format, where Targets might be expected to exaggerate positive aspects of themselves.

It is worth assessing the claim that interview participants’ self-reports of their mental states can be considered as a ground-truth. One objection to using self-reports as ground-truth is that individuals may deliberately misreport their mental states (i.e., lie). While it is always possible that some participants may lie in response to some mental state questions, in the Interview Task the motivation to do so is low. Candidates and Interviewers were taking part in mock job interviews to improve their chances of employment, and as such, providing honest reports of their mental states allows them to receive useful feedback. Similarly, social desirability effects were minimised by the fact that participants acknowledged that their mental state reports would not be seen by anyone other than the researchers. Participants therefore had little reason to conceal any unfavourable beliefs about their counterparts. Furthermore, for several of the mental state questions, it is not clear that one response is more positive than another, meaning these reports should be independent of any social effects. A second objection is that individuals might lack the capacity to report their mental states because they do not know what their mental states are. Intuitively, this objection might be based on the idea that other people (therapists, loved ones) know us better than we know ourselves. However, is difficult to argue that another individual knows our mental states (what we *currently* believe, or *currently* intend) better than we do ourselves because of the privileged access we have to our own consciousness. Indeed, privileged access accounts of self-knowledge, specifically relating to mental states, have long featured in philosophical discourse on this topic^47^.

The third finding, that Mind-space accuracy—i.e., the accuracy with which covariation between personality dimensions is modelled—predicts ability to locate someone in Mind-space replicates the finding of Conway and colleagues^36^. This finding is consistent with the idea that individuals learn to construct their Mind-space through experience of interacting with other people^35^. Individuals who are better able to classify the personality and cognitive abilities of those they encounter have a more accurate sample of trait covariation to be used in constructing a model of how different dimensions of minds (co-)vary. Alternatively, an accurate model of co-variation may lead to more accurate inferences about traits for which diagnostic behaviour is not available, based on information about traits for which diagnostic behaviour is available.

Despite the overall consistency with the results of Conway and colleagues^36^, it is worth considering how reliable mental state inference accuracy is expected to be. It is clear that the Interview Task is not ‘process pure’—performance is not driven by a single cognitive process, and multiple strategies are likely to enable successful performance. These features are generally considered not to be ideal when constructing a cognitive test—preferably one would be able to make a clear inference about a single psychological process on the basis of test performance, and the potential use of multiple strategies makes it difficult to design comprehensive control conditions. Performance on the Interview Task might be based on the linguistic content of the Candidate and Interviewer’s speech, their facial or bodily expressions, the prosody of their voice, reaction time in responding to questions, movement kinematics, or any one of a number of other cues, requiring multiple psychological processes or combinations of processes to be utilised effectively in inferring mental states. However, if one defines individual differences in ToM as differences in the accuracy of mental state inference (rather than, for example, propensity to attribute mental states), multiple psychological processes must contribute to performance, especially if at least a degree of ecological validity is desired. It is therefore possible that a great deal of within-subject variability in the accuracy of mental state inference might exist. Individuals who rely more on prosody and tone of voice when inferring mental states may be likely to be disproportionally affected by text-based conversation, leading to a greater reduction in accuracy of mental state inference than those that rely on semantic cues. Similarly, individuals relying more on facial and bodily expressions of emotion might suffer a drop in the accuracy of mental state inference when using the telephone compared to face-to-face conversations, that is greater than the drop in accuracy experienced by those relying on information derived from the voice. As well as mental state inference accuracy varying as a function of the type of information available, it has previously been speculated that mental state inference accuracy may vary depending on Target characteristics, including their group membership^48,49^ or the degree to which they are similar to the individual inferring their mental states^50,51^. Indeed, Conway et al.^36^ showed that individuals were better able to locate others in Mind-space who were similar to themselves, and the current data and those of Conway and colleagues show that individuals are better able to infer the mental states of Targets that they can accurately locate in Mind-space. As such, it is an empirical question as to whether one can even speak of a general ‘ToM ability’. This should be considered if attempting to assess the test–retest reliability of tasks like that described here. Without multiple types of test (all with ground-truth data) which both vary in the information available to use in mental state inference and in Target minds, it is unclear whether individuals are reliable in the accuracy of their mental state inferences.

Conversely, there are reasons to hypothesise a degree of reliability in the accuracy of mental state inferences. As detailed in the Introduction, under the Mind-space framework sources of individual differences in mental state inference accuracy are due to: (1) The accuracy of Mind-space (particularly of any covariance between dimensions); (2) The ability to locate accurately a Target mind within Mind-space; (3) The ability to combine diagnostic information about the situation and the location of the Target in Mind-space to infer accurately their mental state; (4) The propensity to consider position in Mind-space before making a mental state inference. Both factors 1 and 4, the accuracy of Mind-space and the propensity to consider location in Mind-space, are likely to be relatively stable across Target individuals and the type of information used to make mental state inferences (though there may be effects of group membership on the propensity to consider location in Mind-space). It may also be the case that individual differences in social attention and learning ability mean that some individuals are consistently good, or bad, at inferring mental states regardless of the cues available to them (i.e., across facial expressions, semantic cues, and kinematics).

It should be acknowledged that there are some limitations of this study, and some questions left unresolved. For example, results showed that updating of a Target’s location in Mind-space predicted updating of mental state inferences. It was predicted that this result would vary depending on the relationship between the dimension upon which the Target’s position was updated and specific mental states. If the Target’s location moved solely on one dimension, then mental states for which that dimension predicted a larger amount of variance would be updated to a greater extent than those with a weaker relationship to that dimension. Although this general pattern was observed (there was a great deal of variation in the relationships between specific Mind-space dimensions and mental states), an independent measure of the participant’s judgement of the relationship between particular dimensions in Mind-space and the likelihood of particular mental states was not obtained. Similarities between participant judgements about trait-mental state relationships and our fitted associations would provide useful further support for this hypothesis, given the lack of an established way to determine the significance of the random effects variances which support this hypothesis in the present study. However, Conway and colleagues^36^ previously showed differences in mental state inferences induced by dimension-specific information using false belief stories, and so it is plausible that the same mechanism was responsible for the current results.

One question yet to be addressed by the Mind-space framework concerns the dynamics of the relationship between mind representation and mental state inference. Whilst the present study demonstrates that inference of traits and mental states are related, it does not reveal the origin of this relationship. The dynamics of the relationship are interesting as mental states are momentary, and thus likely to be inferred on the basis of more enduring factors such as traits. However, it is also likely that the feedback which occurs when an estimated mental state is made explicit will induce some level of prediction error, which could in turn be used to update the representation of the Target’s traits. Furthermore, the present study does not allow us to examine the influence of individuals’ propensity to locate Targets in Mind-space on mental state inference accuracy, which we would expect to be a source of variance in mental state inference accuracy in real life. This is because participants were required to make their trait inferences prior to their mental state inferences, and so were made to locate individuals in Mind-space before inferring their mental states.

In summary, a novel task was used to assess ToM, specifically the accuracy of mental state inferences. The use of this task enabled predictions of the Mind-space framework to be tested. Results were in line with predictions, suggesting that representation of a Target’s mind is used when inferring their mental states.

## Supplementary Information

Supplementary Information.

## Data Availability

The datasets generated and/or analysed during the current study are not publicly available due to participant restrictions on data sharing, but shareable data are available from the corresponding author on reasonable request.
