# Supplementary material for: Novel theory of mind task demonstrates representation of minds in mental state inference
Source: Sci Rep. 2022 Dec 7;12:21133. doi: 10.1038/s41598-022-25490-x (PMC9729182; doi:10.1038/s41598-022-25490-x)
Supplement: Supplementary file 1 — Supplementary Information. [file 41598_2022_25490_MOESM1_ESM.pdf]

Novel Theory of Mind task demonstrates representation of minds in mental state inference

Supplementary Information

Emily L. Long*[1], Hélio Clemente Cuve[1], Jane Rebecca Conway[2,3], Caroline Catmur[4], and Geoffrey Bird[1]

[1]Department of Experimental Psychology, University of Oxford, Oxford, OX2 6GG, UK.
[2]Institute for Advanced Study in Toulouse, Université Toulouse 1 Capitole, 31080 Toulouse Cedex 06, France.
[3]School of Psychology, University of Galway, Galway, H91 TK33, Ireland.
[4]Department of Psychology, Institute of Psychiatry, Psychology & Neuroscience, King's College London, De Crespigny Park, Denmark Hill, London SE5 8AF, UK.

Corresponding author: Emily L. Long, emily.long@psy.ox.ac.uk, Department of Experimental Psychology, Anna Watts Building, Woodstock Rd, Oxford OX2 6GG, UK

S1. Mental state questions

S1.1.

The following questions were those answered in private by Targets when reporting their mental states. The questions are given in regular text whilst the labels of the slider ends are given in ***bold.*** *All questions were included in analyses of mental state inference.*

Candidate

1. How would you rate your performance in the interview? **Very poor – Excellent**
2. How engaging have you been to the interviewer? **Not at all engaging – Very engaging**
3. Did you seem confident to the interviewer? **Not at all confident – Very confident**
4. Did you seem competent to the interviewer? **Not at all competent – Very competent**
5. Did you seem experienced to the interviewer? **Not at all experienced – Very experienced**
6. Did you seem emotionally intelligent to the interviewer? **Not at all emotionally intelligent – Very emotionally intelligent**
7. Are you more confident in your interpersonal or your technical skills? **Interpersonal - Technical**
8. What do you think of the interviewer? **Very unfriendly – Very friendly**
9. Did the interviewer extract information well? **Not at all well – Very well**
10. Did the interviewer put you at ease? **Not at all at ease – Very at ease**
11. Did the interviewer seem attentive? **Not at all attentive – Very attentive**
12. How nervous does the interviewer think you are? **Not at all nervous – Very nervous**
13. How confident are you that you would get the job? **Not at all confident – Very confident**
14. To what extent do you think the interviewer is:
    a. **Not at all honest and humble – Very honest and humble**
    b. **Not at all extraverted – Very extraverted**
    c. **Not at all agreeable – Very agreeable**
    d. **Not at all conscientious – Very conscientious**
    e. **Not at all open to experience – Very open to experience**
    f. **Not at all emotional – Very emotional**
15. To what extent do you think the interviewer feels:
    a. **Not at all joyful – Very joyful**
    b. **Not at all fearful – Very fearful**
    c. **Not at all sad – Very sad**
    d. **Not at all disgusted – Very disgusted**
    e. **Not at all angry – Very angry**

Interviewer

1. How would you rate the candidate's performance in the interview? **Very poor – Excellent**
2. Was the candidate engaging to you? **Not at all engaging – Very engaging**
3. Did the candidate seem confident to you? **Not at all confident – Very confident**
4. Did the candidate seem competent to you? **Not at all competent – Very competent**
5. Did the candidate seem experienced to you? **Not at all experienced – Very experienced**
6. Did the candidate seem emotionally intelligent to you? **Not at all emotionally intelligent – Very emotionally intelligent**
7. Do you think the candidate is more confident in their interpersonal or technical skills? **Interpersonal – Technical**
8. What kind of impression do you think the candidate has of you? **Very unfriendly – Very friendly**
9. Did you extract information well? **Not at all well – Very well**
10. Did you put the candidate at ease? **Not at all at ease – Very at ease**
11. Did you seem attentive? **Not at all attentive – Very attentive**
12. To what extent do you think the candidate is nervous? **Not at all nervous – Very nervous**
13. How confident do you think the candidate is that they would get the job? **Not at all confident – Very confident**
14. To what extent do you think the candidate is:
    a. **Not at all honest and humble – Very honest and humble**
    b. **Not at all extraverted – Very extraverted**
    c. **Not at all agreeable – Very agreeable**
    d. **Not at all conscientious – Very conscientious**
    e. **Not at all open to experience – Very open to experience**
    f. **Not at all emotional – Very emotional**
15. To what extent do you think the candidate feels:
    a. **Not at all joyful – Very joyful**
    b. **Not at all fearful – Very fearful**
    c. **Not at all sad – Very sad**
    d. **Not at all disgusted – Very disgusted**
    e. **Not at all angry – Very angry**

S1.2.

The following questions were those answered by participants when inferring Targets' mental states. The questions are given in regular text whilst the labels of the slider ends are given in ***bold.*** *All questions were included in analyses of mental state inference.*

Candidate general thoughts

1. How would the candidate rate *their* performance in the interview? **Very poor - Excellent**
2. Does the candidate think that *they* seem the following to the interviewer:
    a. **Not at all engaging – Very engaging**
    b. **Not at all confident – Very confident**
    c. **Not at all competent – Very competent**
    d. **Not at all experienced – Very experienced**
    e. **Not at all emotionally intelligent – Very emotionally intelligent**
3. Is the candidate more confident in *their* interpersonal or technical skills? **Interpersonal - Technical**
4. What does the candidate think of the **interviewer**? **Very unfriendly – Very friendly**
5. Does the candidate think that **the interviewer** extracted information well? **Not at all well – Very well**
6. Does the candidate think that the **interviewer** put them at ease? **Not at all at ease – Very at ease**
8. Does the candidate think that the **interviewer** seemed attentive? **Not at all attentive – Very attentive**
8. To what extent does the candidate think that the **interviewer** thinks they are nervous? **Not at all nervous – Very nervous**
9. How confident is the candidate that *they* will get the job? **Not at all confident – Very confident**

Candidate thoughts about interviewer personality

10. Does the candidate think that the **interviewer** is:
    a. **Not at all honest and humble – Very honest and humble**
    b. **Not at all extraverted – Very extraverted**
    c. **Not at all agreeable – Very agreeable**
    d. **Not at all conscientious – Very conscientious**
    e. **Not at all open to experience – Very open to experience**
    f. **Not at all emotional – Very emotional**

Candidate thoughts about interviewer emotions

11. Does the candidate think that the **interviewer** feels:
    a. **Not at all joyful – Very joyful**
    b. **Not at all fearful – Very fearful**
    c. **Not at all sad – Very sad**
    d. **Not at all disgusted – Very disgusted**
    e. **Not at all angry – Very angry**

Interviewer general thoughts

1. How would the interviewer rate the *candidate's* performance in the interview? **Very poor - Excellent**
2. Does the interviewer think the *candidate* is:
   a. **Not at all engaging – Very engaging**
   b. **Not at all confident – Very confident**
   c. **Not at all competent – Very competent**
   d. **Not at all experienced – Very experienced**
   e. **Not at all emotionally intelligent – Very emotionally intelligent**
3. Does the interviewer think that the *candidate* is more confident in their interpersonal or technical skills? **Interpersonal - Technical**
4. What kind of impression does the **interviewer** think that the *candidate* has of them? **Very unfriendly – Very friendly**
5. Does the interviewer think that **they** extracted information well? **Not at all well – Very well**
6. Does the interviewer think that **they** put the candidate at ease? **Not at all at ease – Very at ease**
7. Does the interviewer think that **they** seemed attentive? **Not at all attentive – Very attentive**
8. To what extent does the interviewer think that the *candidate* is nervous? **Not at all nervous – Very nervous**
9. How confident does the interviewer think the *candidate* is that they will get the job? **Not at all confident – Very confident**

Interviewer thoughts about candidate personality

10. Does the interviewer think that the *candidate* is:
    a. **Not at all honest and humble – Very honest and humble**
    b. **Not at all extraverted – Very extraverted**
    c. **Not at all agreeable – Very agreeable**
    d. **Not at all conscientious – Very conscientious**
    e. **Not at all open to experience – Very open to experience**
    f. **Not at all emotional – Very emotional**

Interviewer thoughts about candidate emotions

11. Does the interviewer think that the *candidate* feels:
    a. **Not at all joyful – Very joyful**
    b. **Not at all fearful – Very fearful**
    c. **Not at all sad – Very sad**
    d. **Not at all disgusted – Very disgusted**
    e. **Not at all angry – Very angry**

S2. Power analysis

To our knowledge, there exists no standard method for performing power analyses for the type of analyses used in the present study, in which random effects form an important component of the analysis. Therefore, we decided to obtain an opportunity sample to maximise power for the present study within funding constraints available to the project. However, following Kumle, Võ, and Draschkow (2021), we assessed the power of our study through a simulation-based estimation approach using an independent dataset (N = 25) obtained for another study, in which participants completed a version of the Interview Task.

The independent dataset does not allow for analysis of updates, as these participants only viewed one video for each pair of Targets. We fitted our most complex accuracy model (Acc.3) to this dataset. Then we used the powerCurve function from the simr package (Green & MacLeod, 2016) to test the power to detect the random slope of trait error on the trial grouping term (i.e., to find that model Acc.3 is significantly better fitting than model Acc.2, when using a log likelihood test). The function iteratively generates random data on the basis of the fitted model parameters and tests, for varying numbers of observations per participant or observations per trial, on how many occasions out of 1000 iterations the model of interest outperforms the less complex model. Power curves are given below (Figure S.1.). Every one of the 1000 iterations found that, with the number of observations per participant used in the present study and 50 participants, the inclusion of the trait error random slope on the trial grouping term significantly improved model fit, suggesting that the power of this analysis is greater than 99% (95% CI [99.63, 100.00]).

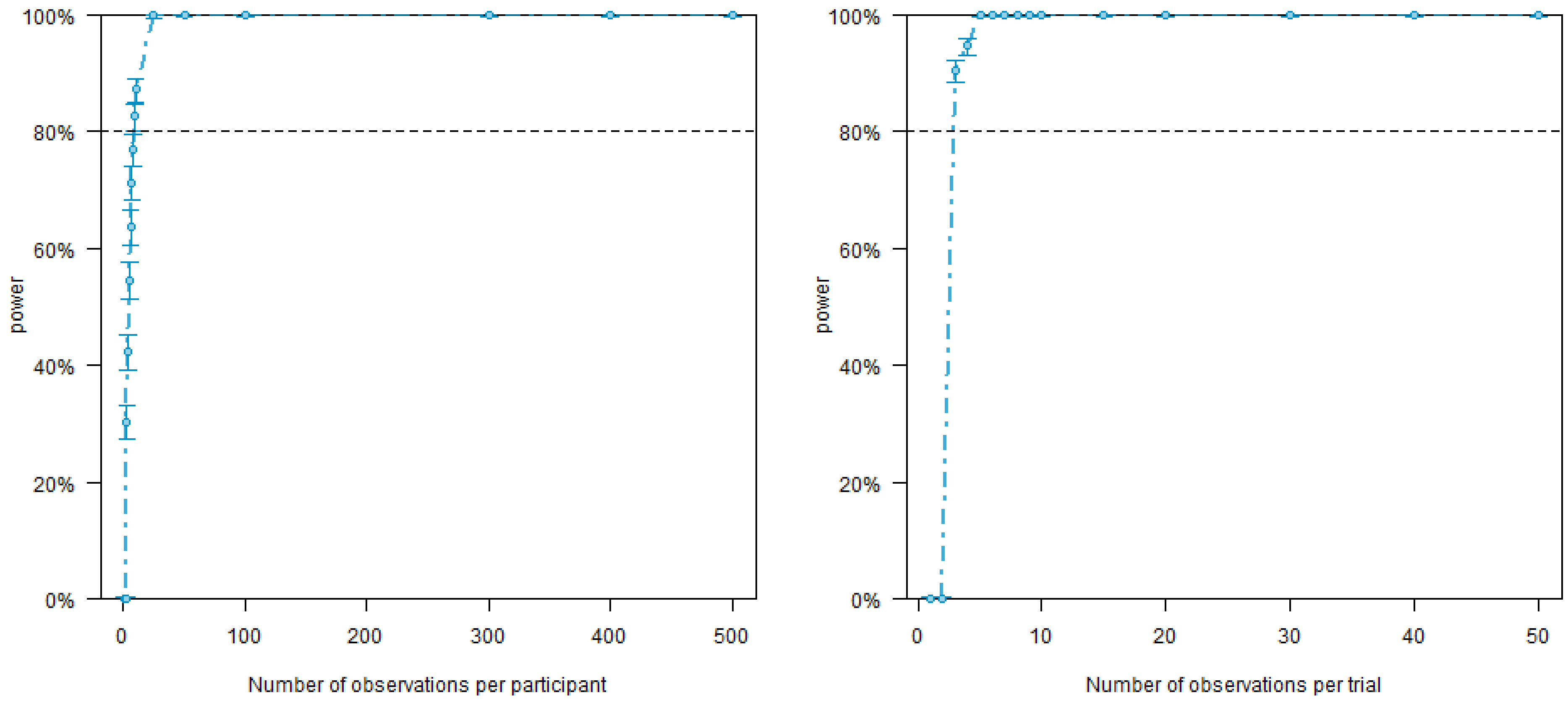

Figure S.1. Power curves from simulation-based power analysis. The curve on the left shows the increase in power with additional observations per participant (trials), suggesting that with 25 participants, nine trials per participant are needed for 80% power to detect the trait error random slope on the trial grouping term. The curve on the right shows the increase in power with additional observations per trial (participants), suggesting that with 576 trials, three participants are needed for 80% power to detect the trait error random slope on the trial grouping term.

Table S.1. Results from model Acc.3 in an independent dataset

**Random effects**			
Groups	Term	SD	Corr
Trial	Intercept	83.68	
	Trait error	22.91	-.16
Participant	Intercept	24.78	
	Trait error	0.77	.60
Residual		521.97	

**Fixed effects**	
Term	Estimate
Intercept	-2.39
Trait error	1.16

Note. These values were used to produce simulated data for power analysis.

S3. Model selection process

For each research question, we included random effects, allowing for both random intercepts and slopes. This allowed us to account for variance in intercepts and slopes across different participants. It also allowed us to examine variance in intercepts and slopes across different trait – target (i.e., Candidate or Interviewer) – mental state combinations. First a null model (X.n) was specified for each question without the fixed effect (predictor) but including participant as a random effect. Next, models with the predictor as a fixed effect (X.1) were compared to the null models. Then, the random slope for the predictor on the participant grouping term was included (X.2) and these models were compared to the previous models (X.1). Then, the additional random effect of trial (trait – target – mental state) was included and these models (X.3) were compared to the models that had been fitted without that random effect (X.2). The final model (X.3) included the predictor of interest both as a fixed effect and a random slope on the trial grouping term and (if justified by the X.2-X.1 comparison) the participant grouping term. All models were checked for degeneracy and this check revealed that both intercepts and slopes explained unique variance, meaning that the maximal models were justified.

Table S.2. – All regression models

Model	Predictor	Random Effects	Outcome	*B*	*SE*	*t*	*p*
SType.n	1	1 | Participant;<br>1 | Trait	Absolute trait update	0.45	0.01	33.41	<.001
SType.1	Trial type	1 | Participant;<br>1 | Trait	Absolute trait update	-0.01 / 0.03	0.01/ 0.02	-1.00/<br>1.77	.317/<br>.077
SType.2	Trial type	1 + Trial type | Participant;<br>1 + Trial type | Trait	Absolute trait update	This model failed to converge and the trial type slope was degenerate for both intercepts.			
SType.3*	Trial type *<br>Time	1 | Participant;<br>1 | Trait	Absolute trait update	Time: *B* = 0.15, *SE* = 0.02, *t* = 7.14, *p* <.001, all other *p*s > .052.			
SType.4	Trial type *<br>Time	1 + Time | Participant;<br>1 + Time | Trait	Absolute trait update	See section S4			
Update.n	1	1 | Participant	MS update	-1.35	0.20	-6.76	<.001
Update.1	Trait update	1 | Participant	MS update	1.19	0.05	23.64	< .001
Update.2	Trait update	1 + Trait update | Participant	MS update	1.21	0.19	6.32	<.001
Update.3	Trait update	1 + Trait update |Participant;<br>1 + Trait update |Trial	MS update	1.20	0.21	5.58	<.001
Acc.n	1	1 | Participant	MS error	-4.92	0.39	-12.55	<.001
Acc.1	Trait error	1 | Participant	MS error	0.82	0.06	12.73	<.001
Acc.2	Trait error	1 + Trait error | Participant	MS error	0.78	0.15	5.07	<.001
Acc.3	Trait error	1 + Trait error | Participant;<br>1 + Trait error | Trial	MS error	0.89	0.29	3.09	.002
PPT.n	1	1 | Participant	Absolute trait error	0.79	0.02	39.49	<.001
PPT.1	PPT (centred)	1 | Participant	Absolute trait error	0.53	0.20	2.63	.011
PPT.2	PPT (centred)	1 | Participant;<br>1 + PPT | Trait	Absolute trait error	0.52	0.21	2.47	.017

Note. n = null model. Updates are calculated as the difference in judgements midway – pre-video, or end – midway. Error values are calculated as the difference between participant inference and ground-truth value and were obtained from the judgements made midway through video and at the end: pre-video judgements were disregarded due to the mismatching intervention. All update or error values are directional unless stated as absolute. Values for the trial type models are given as half / mismatched as the matched type was used as the reference level. PPT = Personality Pairs Task error score. *Note that model SType.3 was compared to model SType.1, not SType.2, because the former outperformed the latter.

Table S.3. – Model comparisons

Model	*AIC*	*BIC*	*LogLik*	*X^2(df)*	*p*
SType.n	4100.2	4126.1	-2046.1		
SType.1	4095.0	4133.7	-2041.5	9.26 (2)	.010
SType.2	4170.3	4273.6	-2069.2	0 (10)	1
SType.3*	3957.8	4015.9	-1969.9	143.21 (3)	<.001
SType.4	3948.3	4032.2	-1961.2	17.48 (4)	.002
Update.n	1632313	1632344	-816154		
Update.1	1631757	1631798	-815875	557.86 (1)	< .001
Update.2	1631203	1631264	-815595	558.63 (2)	<.001
Update.3	1627727	1627819	-813855	3481.39 (3)	<.001
Acc.n	2325199	2325230	-1162596		
Acc.1	2325039	2325080	-1162515	161.84 (1)	<.001
Acc.2	2324882	2324944	-1162435	160.83 (2)	<.001
Acc.3	2196838	2196932	-1098410	128049.63 (3)	<.001
PPT.n	8645.7	8665.3	-4319.8		
PPT.1	8641.2	8667.4	-4316.6	6.51 (1)	.011
PPT.2	8457.4	8503.2	-4221.7	189.79 (3)	<.001

Note. n = null model. SType = trial type update models. Update = update models. Acc = accuracy models. Models are described in Table S.2. *Note that model SType.3 was compared to model SType.1, not SType.2, because the former outperformed the latter.

S4. Trial type update model output

Table S.4. – Trial type update model output

**Random effects**				
Groups	Term	Variance	SD	Corr
Trait	Intercept	0.0006	0.02	
	Time	0.0008	0.03	-.49
Participant	Intercept	0.0010	0.10	
	Time	0.0063	0.08	-.78
Residual		0.13	0.36	

**Fixed effects**					
Term	Estimate	SE	df	t-value	p
Intercept	0.37	0.02	105.79	17.52	<.001
Type Half	0.00	0.02	4614.69	0.27	.788
Type Mismatched	0.04	0.02	4616.10	1.71	.088
Time Pre-mid	0.15	0.03	105.62	5.85	<.001
Half:Pre-mid	-0.03	0.03	4604.76	-1.30	.195
Mismatch:Pre-mid	-0.02	0.03	4605.02	-0.55	.581

S5. IQ modelling

The IQ model selection process followed the same procedure as that used for our primary analyses, outlined in section S3. Here, trial was defined as a unique combination of the target of IQ estimate (Interviewer or Candidate) and the mental state question being answered; this is due to the fact that trait could not vary in this analysis, as IQ was the only trait considered. Whilst no fixed effect of IQ was observed in either of the final models (see tables S.7. and S.8.), the fact that an effect of IQ was observed when the random effect of trial was not included (see table S.5.) and that the random effect of trial significantly improved model fit (see table S.6.), suggests that IQ estimates (and their accuracy), do affect mental state estimates, but that this effect differs across mental states and targets. The null fixed effect result implies that there is no *general* tendency for a single directional effect of IQ, suggesting that effects of IQ work in opposite directions for different mental states and these effects 'cancel out'. These results therefore support our primary analyses and suggest that traits of mind other than personality are also used in mental state inference. Note that IQ values (both errors and updates) were included as z-scores within the model due to convergence issues. The z-score was calculated within target type – i.e., the z-score reflects the extent of the error/update within the category of Candidate or the category of Interviewer. Observations were grouped in this manner before z-scoring due to a general tendency to underestimate Candidate IQs and overestimate Interviewer IQs within our participants.

Table S.5. – All IQ regression models

Model	Predictor	Random Effects	Outcome	*B*	*SE*	*t*	*p*
IQUpdate.n	1	1 | Participant	MS update	- 1.40	0.17	-8.17	<.001
IQUpdate.1	IQ update (z-score)	1 | Participant	MS update	0.32	0.07	4.43	< .001
IQUpdate.2	IQ update (z-score)	1 + IQ update | Participant	MS update	0.36	0.17	2.14	.038
IQUpdate.3	IQ update (z-score)	1 + IQ update | Participant; 1 + IQ update | Trial	MS update	0.32	0.22	1.48	.141
IQAcc.n	1	1 | Participant	MS error	-4.87	0.40	-12.27	< .001
IQAcc.1	IQ error (z-score)	1 | Participant	MS error	0.39	0.15	2.55	.011
IQAcc.2	IQ error (z-score)	1 + IQ error | Participant	MS error	0.37	0.16	2.30	.025
IQAcc.3*	IQ error (z-score)	1 | Participant; 1 + IQ error | Trial	MS error	0.35	0.88	0.39	.696

Note. All IQ values, including in random slopes, were included in the models as z-scores. Z-scores were calculated within target type (i.e., Interviewer or Candidate). In the case of IQ, trial is defined as the target of the IQ judgement and the mental state question being answered. *Note that model IQAcc.3 was compared to model IQAcc.1, not IQAcc.2, because the former outperformed the latter.

Table S.6. IQ model comparisons

Model	*AIC*	*BIC*	*LogLik*	*X^2(df)*	*p*
IQUpdate.n	265219	265245	- 132607		
IQUpdate.1	265202	265236	- 132597	19.58 (1)	< .001
IQUpdate.2	265117	265167	- 132552	89.23 (2)	< .001
IQUpdate.3	264517	264593	- 132250	605.32 (3)	< .001
IQAcc.n	386550	386576	- 193272		
IQAcc.1	386546	386580	- 193269	6.46 (1)	.011
IQAcc.2	386549	386600	- 193268	0.83 (2)	.659
IQAcc.3*	361565	361625	- 180775	24987.00 (3)	< .001

Note. *Note that model IQAcc.3 was compared to model IQAcc.1, not IQAcc.2, because the former outperformed the latter.

Table S.7. IQ update model output

**Random effects**					
Groups	Term	Variance	SD		
Trial	Intercept	2.70	1.64		
	IQ (z-score)	1.85	1.36		
Participant	Intercept	1.48	1.22		
	IQ (z-score)	1.15	1.07		
Residual		161.11	12.69		
**Fixed effects**					
Term	Estimate	SE	df	t-value	p
Intercept	-1.39	0.25	119.67	-5.66	< .001
IQ (z-score)	0.32	0.22	91.69	1.48	.141

Model formula: Mental state update ~ IQ update (z-score) + (1 + IQ update (z-score) | Participant) + (1 + IQ update (z-score) | Trial)

Note. In the case of IQ, trial is defined as the target of the IQ judgement and the mental state question being answered.

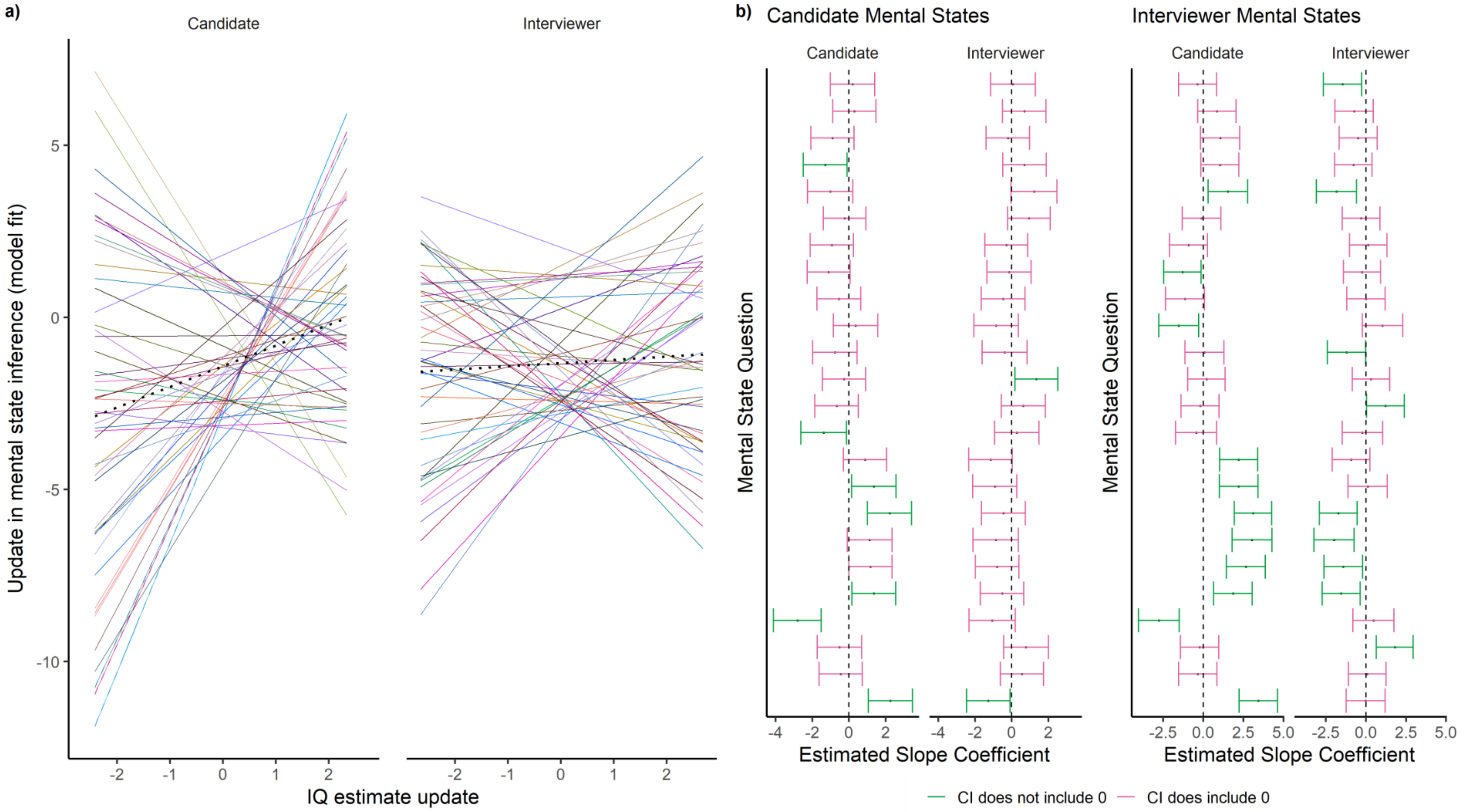

Figure S.2. Plots depicting the relationships between IQ update and mental state updates. Panel a) The specific relationships between updates in IQ estimates for the Candidate (left) and Interviewer (right) and updates in specific mental states (represented by different colours). Panel b) The estimated slopes for these relationships. Error bars are 95% confidence intervals and green lines indicate that the confidence interval does not include zero, while pink lines indicate that it does.

Table S.8. IQ accuracy model output

Random effects

Groups	Term	Variance	SD	Corr
Trial	Intercept	371.93	19.29	
	IQ (z-score)	72.44	8.51	.01
Participant	Intercept	8.26	2.87	
Residual		459.72	21.44	

Fixed effects

Term	Estimate	SE	df	t-value	p
Intercept	-4.49	2.01	103.22	-2.24	.028
IQ (z-score)	0.32	0.88	96.24	0.37	.716

Model formula: Mental state error ~ IQ error (z-score) + (1 | Participant) + (1 + IQ error (z-score) | Trial)

Note. In the case of IQ, trial is defined as the target of the IQ judgement and the mental state question being answered.

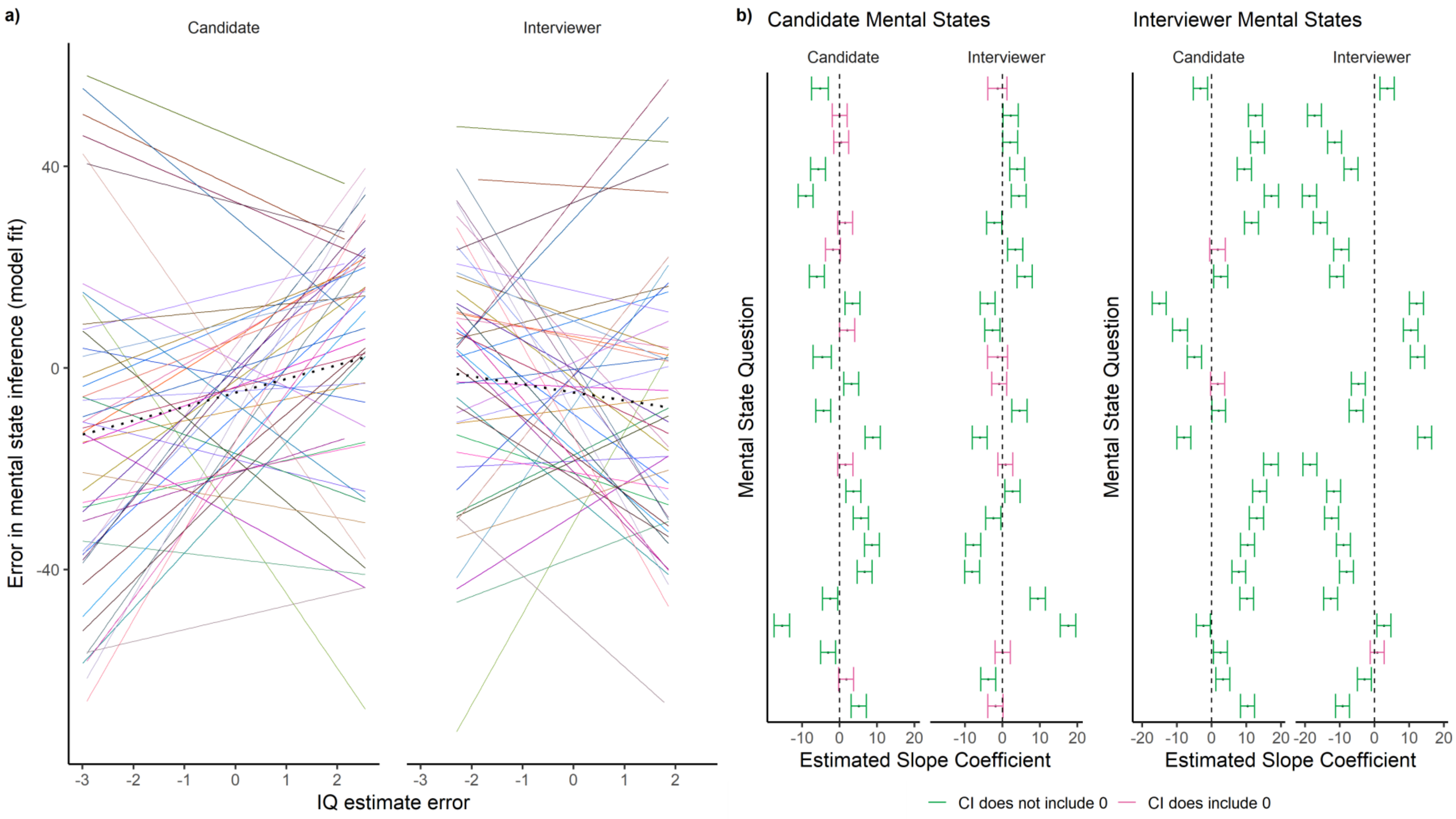

Figure S.3. Plot depicting the relationships between IQ error and mental state error. Panel a) The specific relationships between error in IQ estimates for the Candidate (left) and Interviewer (right) and error in specific mental states (represented by different colours). Panel b) The estimated slopes for these relationships. Error bars are 95% confidence intervals and green lines indicate that the confidence interval does not include zero, while pink lines indicate that it does.

S6. Simulated parameter distributions for model effects (models Acc.3 and Update.3)

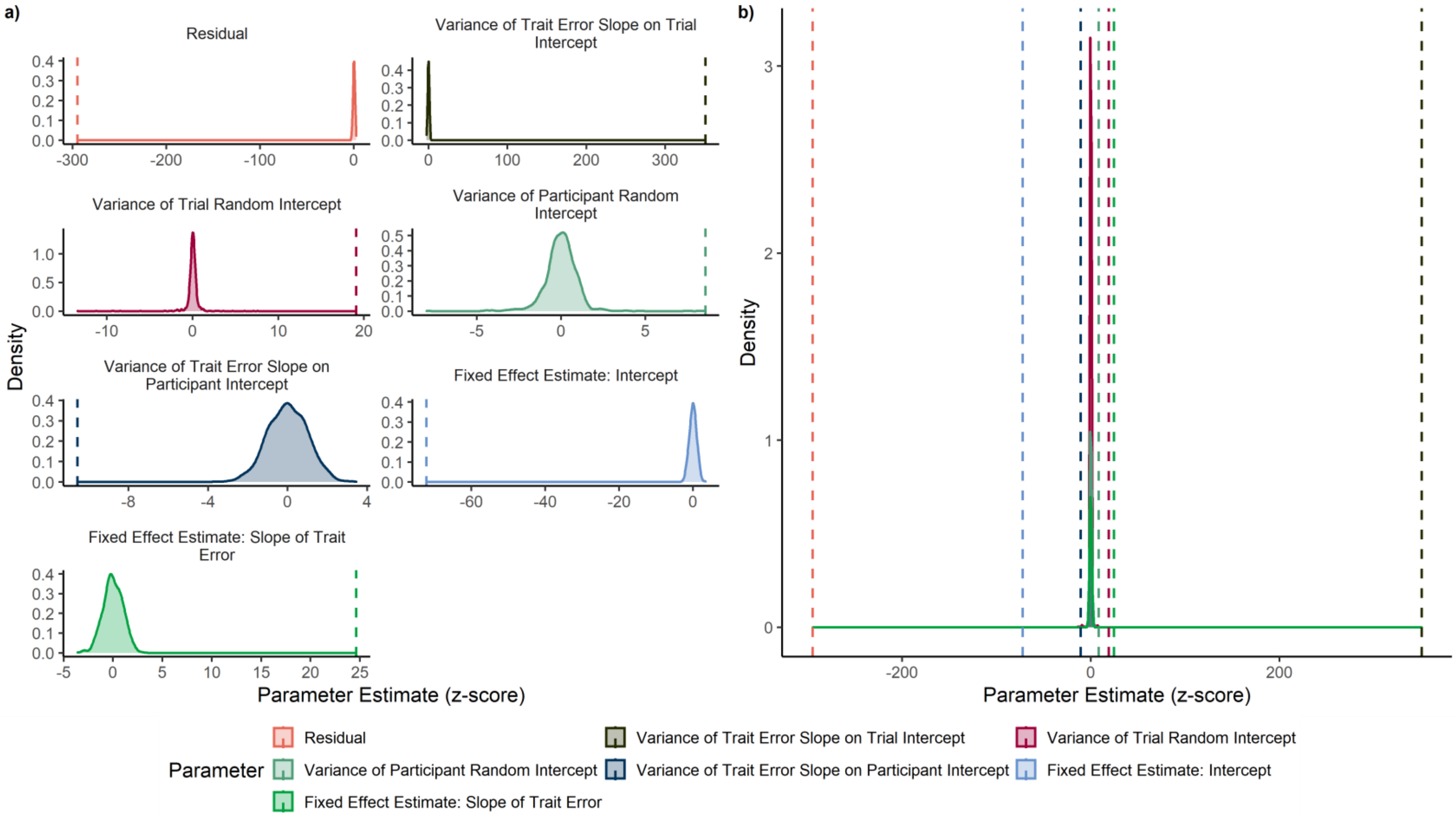

Figure S.4. Histograms showing the null distributions of parameters for model Acc.3, taken from 1000 iterations of shuffling trait error between trait labels, when grouped by participant, stimulus and mental state. Panel a) shows the parameter distributions individually whilst panel b) depicts all parameters together. All values are given as z-scores relative to simulated values of the given parameter. Dotted lines indicate the z-score of our observed value relative to the simulated distribution.

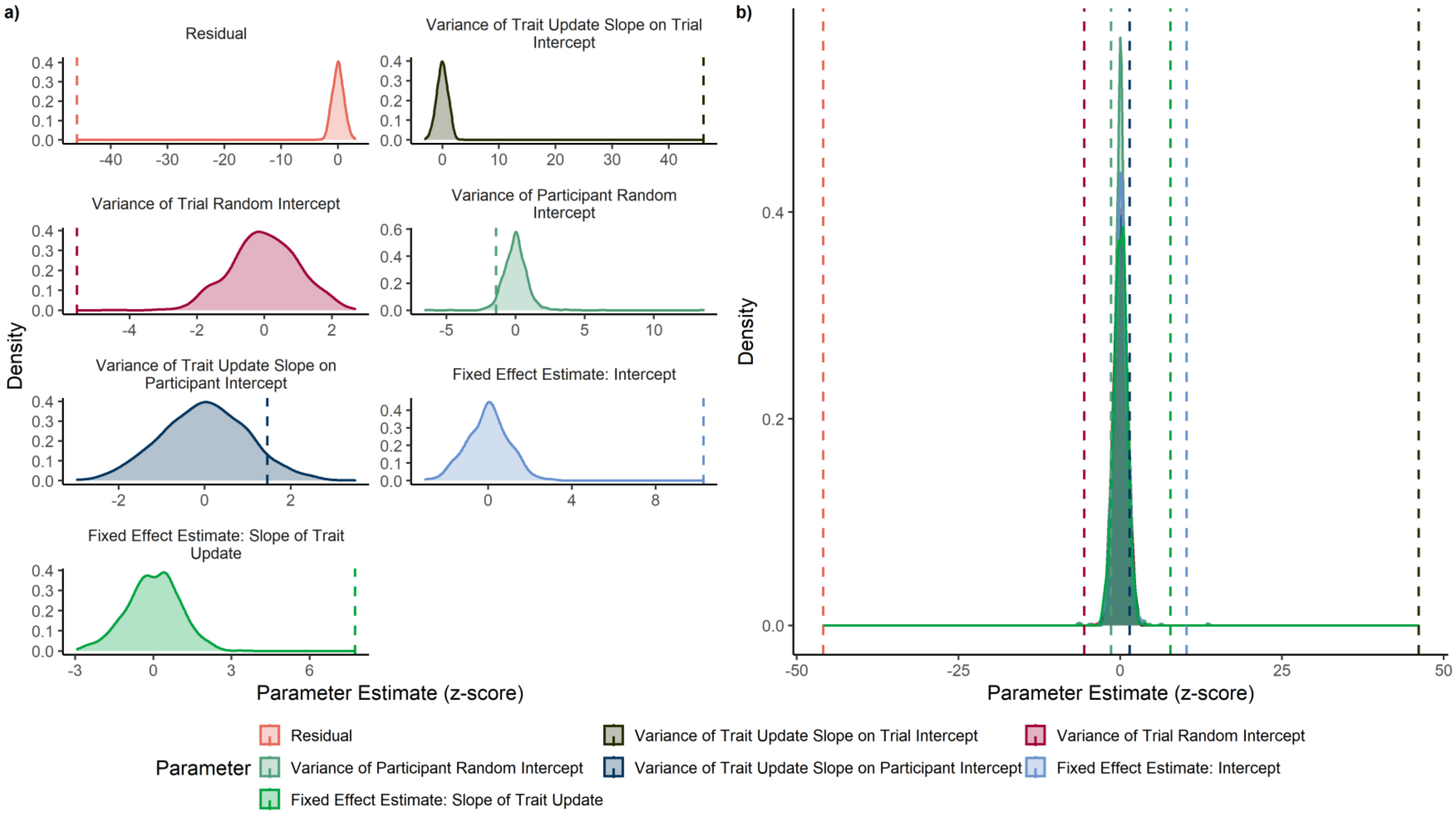

Figure S.5. Histograms showing the null distributions of parameters for model Update.3, taken from 1000 iterations of shuffling trait update between trait labels, when grouped by participant, stimulus and mental state. Panel a) shows the parameter distributions individually whilst panel b) depicts all parameters together. All values are given as z-scores relative to simulated values of the given parameter. Dotted lines indicate the z-score of our observed value relative to the simulated distribution.

References for power analysis (section S.2.)

Green, P., & MacLeod, C. J. (2016). SIMR: an R package for power analysis of generalized linear mixed models by simulation. *Methods in Ecology and Evolution, 7*(4), 493-498. doi:https://doi.org/10.1111/2041-210X.12504

Kumle, L., Võ, M. L. H., & Draschkow, D. (2021). Estimating power in (generalized) linear mixed models: An open introduction and tutorial in R. *Behavior Research Methods, 53*(6), 2528-2543. doi:10.3758/s13428-021-01546-0
